# The impact of interaction on the adoption of electric vehicles: Mediating role of experience value

**DOI:** 10.3389/fpsyg.2023.1129752

**Published:** 2023-02-28

**Authors:** Wenbo Li, Mengzhe Wang, Xiu Cheng, Ruyin Long

**Affiliations:** ^1^Business School, Jiangsu Normal University, Xuzhou, China; ^2^College of Economics and Management, Nanjing Forestry University, Nanjing, China; ^3^School of Business, Jiangnan University, Wuxi, China

**Keywords:** interaction, electric vehicle, purchasing intention, experience value, different business models

## Abstract

The widespread adoption of electric vehicles (EVs) largely depends on the acceptance of the public. Previous studies pay more attention to the factors affecting EV adoption from the customer perspective but lack the perspective of the interaction between sellers and customers. Based on a survey of 1,014 respondents in China, this work developed a research model analyzing the effect of interaction on the intention to purchase EVs and using experience value (EPV) as the mediating variable. The results showed that the functional experience value (FEV) was positively affected by the environment–customer interaction (ECI). The FEV, emotional experience value (EEV), and social experience value (SEV) were all positively affected by salesman–customer interaction (SCI). In addition, they all had positive impacts on purchasing intention (PI). We further analyzed the differences in the interaction between the different business models. Compared with multi-business model car companies, the ECI for single-business model car companies had a more positive impact on the PI. However, the impact of ECI for single-business model companies on PI was negative. The SCI of single-business model car companies positively impacted the PI, whereas the SCI of multi-business model car companies had no significant impact on the PI. These findings provide insight into further understanding the mechanism of interactions affecting EV adoption and help perfect future promotion strategies.

## 1. Introduction

Due to economic expansion and improving living standards, the transportation industry now contributes significantly to worldwide energy consumption and greenhouse gas emissions, i.e., 32 and 25%, respectively (McCollum et al., 2018). In order to achieve the emission reduction goal, Electric vehicles (EVs), mainly pure battery EVs and plug-in hybrid EVs, are becoming increasingly prevalent modes of transport worldwide. Compared with conventional fuel vehicles (CFVs), zero emissions during the EV driving stage can significantly enhance the air quality in cities and mitigate the health risk of tailpipe pollution (Li et al., 2021a,b). Incentive policies to promote EVs have recently been implemented in many countries, such as China, the US, and Europe. These policies mainly comprise financial subsidies (such as vehicle purchase subsidies, purchase tax exemptions, and license fee exemptions) and driving privileges (such as no traffic restrictions and parking fee exemptions). The global EV market is increasing thanks to the above favorable policies; roughly 6 million were sold globally in 2021. However, these sold EVs only account for 9% of total vehicle sales. China has been the world’s largest production and sales market for EVs and the world’s largest EV exporter in 2021. Such achievement in China is attributed to the incentive policies of EVs and the purchase restriction of CFVs (e.g., license plate lottery and license plate auction). By the end of 2021, there were 7.84 million EVs in China, but the EV ownership rate was just 3.23% (IEA, 2022). Thus, the low penetration rate of EVs is a significant problem for EV development. Current EV development relies mainly on external stimulation of subsidy policy, but China’s subsidy policy is about to be canceled in 2023 (Li et al., 2021a,b). Moreover, China is currently striving to achieve the goal of carbon peaking by 2030 and carbon neutrality by 2060. Therefore, exploring new ways to increase customers’ intention to purchase EVs is very crucial.

Previous studies on EV adoption mainly concerned customers’ preferences. Benefits of EVs that attract customers include low daily driving costs, quiet driving, strong acceleration, and incentive policies (Li et al., 2020; Mandys, 2021). On the contrary, attributes that make customers resist EVs include high purchase prices, low mileage, long refueling time, insufficient charging stations, and high maintenance cost (Kumar and Alok, 2020). These studies focused on improving the promotion of EVs based on the perspective of customers, but they ignored the problems with EV sales which is also very important for EV promotion. A few studies showed there are some problems during the EV sales process. Due to less profit, many dealers are not motivated to sell EVs (Gerardo et al., 2018), so the perception of the experience of purchasing EVs is worse than CFVs (Cahill et al., 2014). What is more, salespeople lack sufficient knowledge and skills; sometimes they even convey negative viewpoints about the prospects of EVs to customers. A lack of EV models on site to view or test drive is also a common barrier for customers (Matthews et al., 2017). Presently, customers have limited knowledge about EVs, so going to the car shop is one of the effective ways to help them learn more about the advantages of EVs (Gerardo et al., 2018; Yang et al., 2021). The interaction is critical in promoting EVs because it is the final link before customers purchase EVs. In contrast to the usual emphasis on product quality alone, Hong et al. (2020) noted that customers now prefer to increase their intention to purchase through interactive experiences and emotional resonance. Since the commercial marketing layout of EVs becomes thematic, scenario-based, and overlaying, it is necessary to explore the effect of interaction in EV selling (Yang et al., 2021).

In addition, to satisfy the demand for potential car buyers, social media and apps provided by car companies contribute to improving their EV experience. However, the possible effect of these digital platforms on EV selling has not been thoroughly analyzed. Wamuyu (2018) argued that social media are essential for delivering messages related to the promotion of sustainability and social issues, such as environmental protection. De Fano et al. (2022) found that many companies are increasingly using social media to interact with customers and guide them to green consumption to promote the sustainability activities. However, the innovative digital marketing demands a thorough understanding of customers’ needs to close the gap between actual and desired shopping experiences (Tupikovskaja et al., 2021). Thus, when analyzing the effect of interaction in EV selling, the online interactions between car companies and customers provided by social media and apps need to be considered.

With the EV market’s development, interactions in EV selling differ between companies. Business models of EV companies can be divided into two catalogs: multi-business model car companies and single-business model car companies. The former sells both EVs and CFVs, and the latter sells only EVs. Emerging Chinese car companies in China, such as NIO and Xiaopeng, which are single-business model car companies, have developed new selling methods based on purchasing EVs online and experiencing EVs offline. They have also introduced more scenario-based experiences in offline sales stores (Yang et al., 2021). These sales methods provide more added value for EVs, cater to customers’ demands in the era of the experience economy, and achieve excellent results in EV promotion. By contrast, most traditional car companies, which are multi-business model car companies, still follow the traditional 4S store sales method. They have some problems in the sale of EVs and need to improve in sales motivation. Thus, the difference analysis between business models can help better understand the effect of interactions.

This study aims to analyze the effect of interactions on the intention to purchase EVs from the perspective of different business models. Compared with previous studies, the contributions of this study are summarized as follows. First, we defined the interactions in EV selling and highlighted the effect of online interactions on different experience values. Second, we extended the theory of stimulus–organism–response (SOR) model by considering the interactions. Third, we considered the difference in interactions for car companies with multi-business and single-business models. These findings provide new insights for improving customers’ intention to purchase EVs and give a reference for formulating EV promotion strategies by policymakers.

The rest of this paper is organized as follows: the conceptual model and hypotheses are presented in the next section; the third section introduces the methods and data sources; the results are presented and discussed in the fourth and fifth sections; and the last section concludes the paper.

## 2. Conceptual model and hypotheses

### 2.1. Research variables

#### 2.1.1. Definition of interaction

In early studies, interactions in selling goods are defined as the communication between customers and sellers. Later, customer interactions with the environment and other customers are gradually considered. Svensson (2001) contended that interaction is more than a simple surface-level phenomenon; it requires many components (including interpersonal interaction, environmental interaction, etc.) to be expressed and measured. Li and Fan (2006) proposed an extended interaction model presenting the service interactions between customers and companies, including customers, employees, systems, and physical environments. Chen et al. (2022) categorized interactions during service into three types: communication and interactions between customers and sellers, the environment, and items. In EVs selling, interactions mainly occur between the customer and the environment and between the customer and the salespeople. Specifically, customers interact with the EV environment by looking at the cars in physical stores and browsing EV-related information online. Interactions between the customer and salespeople include discussing EVs and the test drive accompanied by the salespeople. Based on the above, interactions investigated in this study include environment–customer interaction (ECI) and salesman–customer interaction (SCI).

The ECI in previous studies was limited to an offline environment. As the efficiency of interactions was enhanced by technology, Bitner (1992) proposed that when exploring an environment, it should include not only offline visible elements but also online platforms’ technological elements, such as electronic services. Thus, in this study, ECI was further classified into online ECI and offline ECI. Online ECI includes customer perceptions of the convenience and esthetics of digital platforms such as apps and websites launched by car companies and the allure of third-party social media for potential EV buyers. Offline ECI describes how the ambiance established by offline EV storefronts might affect customers’ views of EVs (Singh and Prashar, 2014). This ambiance includes smell, lighting, facility presentation, cleanliness, and location. Product interaction (PD) and verbal interaction (VB) are two categories of SCI. PD refers to the customer’s entire understanding of EVs’ efficiency, convenience, and sophistication during the test drive accompanied by the salespeople. VB includes the professionalism, demeanor, and sales skills of the salespeople, as well as the customer’s questions, needs, and feedback based on the description of the EV’s performance.

It is important to note that both ECI and SCI involve indicators from two distinct categories. The removal of any one indicator would change the concept of the two variables (Jarvis et al., 2003). In reality, customers do not need to engage in all kinds of interactions, because these interactions are not within a common theme and are not highly associated. Specifically, customers might not interact with car companies online, including not using an app or reading social media posts. In addition, there may be no PD between the customer and the salespeople, i.e., only VB and no test drives. So, the characteristics of ECI and SCI were consistent with the concept of formative variables. In other words, the indicators that comprise the formative variable do not necessarily have identical content and cannot be substituted for each other. Therefore, in this study, ECI and SCI were regarded as second-order formative variables based on the conceptual properties of formative variables.

#### 2.1.2. Definition of experience value

In the age of experience economies, customer desires are progressively turning toward the spiritual sphere. This change expands and enhances experience theory, resulting in the concept of experience progressively arising in the context of products. Trischler et al. (2018) defined customer experience as a customer journey involving multiple touchpoints and interactions with different actors. Experience provides sensory, emotional, cognitive, behavioral, and relational values. Customers desire and seek experience because experience value is found in the experience and can endure for a long time (Pine and Gilmore, 2013). Sweeney and Soutar (2001) contended that customers’ needs in some industries are hierarchical, and thus scholars can establish the correspondence between different needs of customers and different experience values from the vertical, so as to realize hierarchical experience values based on customers’ needs. Based on the research on customer experience in the service industry, Li and Fan (2006) uphold Sweeney’s perspective and commented that consumers are both emotional and rational, and they require the satisfaction of psychological needs and a sense of belonging while pursuing physical basic needs. Thus, they believed that service experience can be divided into three dimensions: functional, social, and emotional. The hierarchical division method corresponds the experience value to the five levels of needs: physiological needs, security needs, belonging needs, respect needs, and self-actualization, and believes that customers experience satisfaction because the needs at different levels are satisfied (Sweeney and Soutar, 2001). As a result, the hierarchical division method can more accurately reflect the experience value.

Therefore, in this study, we categorized experience value (EPV) as functional experience value (FEV), emotional experience value (EEV), and social experience value (SEV). The FEV corresponds to the satisfaction of customers’ functional needs, including the efficiency or convenience of obtaining EV information (Shen et al., 2016). Specific to EVs, FEV is the customer’s understanding of performance attributes, development prospects, and EV policy trends during the interaction. EEV refers to the emotions generated during the EV interaction, particularly in identification with the interaction and the ongoing focus on EVs. It specifically refers to customers’ feelings about the enthusiasm and sincerity of salespeople, the technical skill of salespeople in selling EVs, and the overall satisfaction with the interactive process. SEV means that customers can get respect and social identity and show their values during the interaction. It refers explicitly to customers believing that purchasing an EV is a green consumption decision, and this behavior can enhance their social image and gain more social recognition (Higueras-Castillo et al., 2019).

### 2.2. Conceptual model

**Figure 1:**
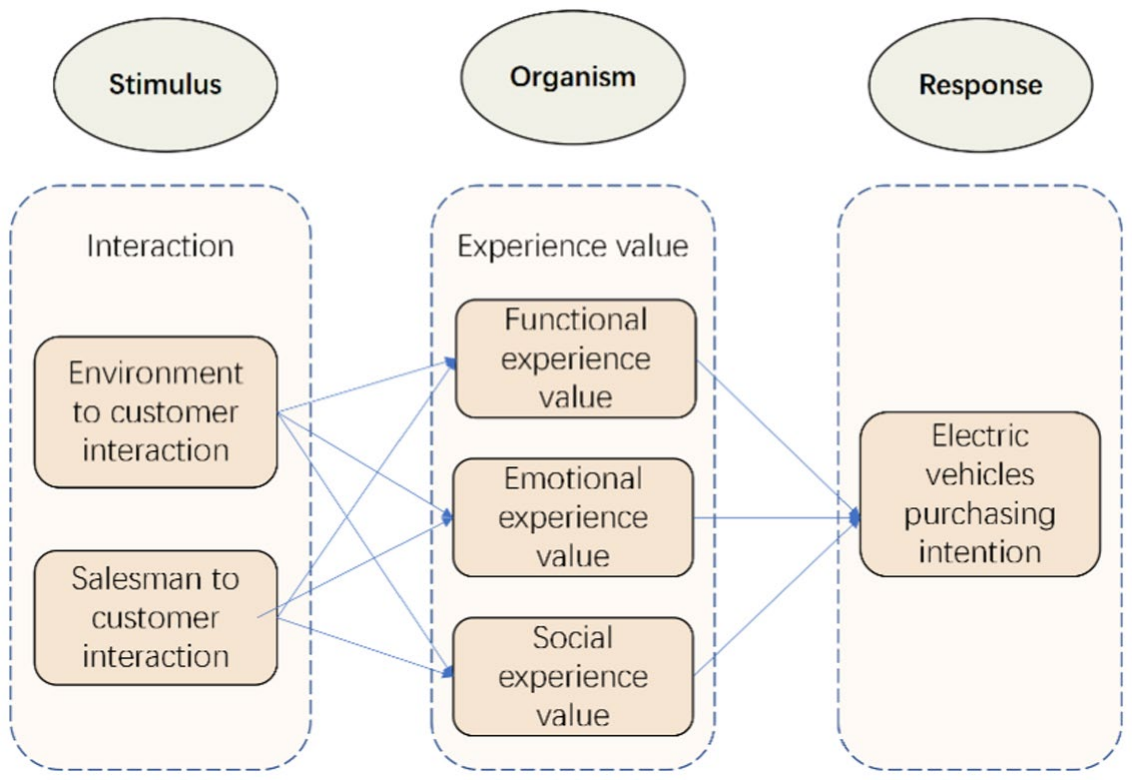
Conceptual model.

Unlike the Input–Output theory, the expanded SOR (Stimulus-Organism-Response) theory does not discount the significance of intraindividual factors. It adds variables that can express internal perceptions and changes in psychological factors. SOR theory assumes that environmental stimuli cause an individual to experience emotional or cognitive changes, which then affect individuals’ subsequent attitudes and behavioral reactions. Therefore, one of the preferred theoretical models for researching customer behavior is the SOR model. In order to understand why customers choose to purchase green products, Wang (2017) built a SOR model based on external risk as a stimulus and customer purchasing intention as a mediating variable. Tak and Gupta (2021) used the SOR model to examine the role of customer engagement in travel mobile device attributes on the customer using intention. The SOR theory assumes that each interaction during a sales process has the potential to enhance, deteriorate, or even destroy a customer’s EPV. The EPV may further affect the customers’ perception of EVs’ performance attributes and service quality, their intention to purchase an EV in this company, and their subsequent purchasing behavior. During the EV selling process, the interactions between car companies with various business models and customers can be understood using interaction theory. Previous studies have demonstrated that interactions can substantially affect individual EPV, which could further affect the purchasing decisions of customers (Shen et al., 2016). Therefore, in this study, we considered that external stimulus referred to interactions, organism referred to the EPV produced during the interactions, and response referred to the customer’s intention to purchase EVs. We propose a conceptual model of the effects of interactions on the intention to purchase EVs, as shown in Figure 1.

### 2.3. Hypotheses

#### 2.3.1. Impact of interactions on the EPV

1. Impact of ECI on EPV

The vehicle layout, lighting, scent, and interior displays in sales stores are classified as the interaction environment (Bitner, 1992), and they theoretically contribute to the EPV. Many studies have confirmed that environmental elements significantly affect customers’ emotions and purchasing behavior. In addition, Pantano et al. (2021) investigated the impact of time convenience on customer patronage behavior. They showed that time convenience was a determinant of customer choice for shopping location. One of the primary services offered by businesses should be time utility or offering interactions at the appropriate time and location (Gulzari et al., 2022).

In addition to the offline ECI, online ECI also affects the customer experience. Due to their positive effects on the information sharing, brand recognition, and individual engagement, online social media platforms influence every aspect of consumer decision-making (Cao et al., 2021). Companies are gradually developing digital platforms for customer involvement in sustainable behavior (Japutra et al., 2021). According to Lemon, companies that communicate with customers more frequently on digital platforms can affect the customer experience. As a result, ECI no longer occurs only in physical stores; instead, they are more prevalent online. Combining digital and online technologies, these car companies launch digital platforms with unique page layouts, background music, novel short films, and comprehensive services. These measures create a comfortable and harmonious atmosphere and attract customers to immerse themselves in browsing the pages, thus enhancing their EEV (Pantano et al., 2021). At the same time, the more frequently customers interact with online environmental services, the more knowledge and skills they learn about EVs. Thus, the following hypotheses were addressed in this study.

*H1a*: ECI has a positive effect on FEV.

*H1b*: ECI has a positive effect on EEV.

*H1c*: ECI has a positive effect on SEV.

1. Impact of SCI on EPV.

In the study of customer service, Groth et al. (2019) found that interaction is central to the experience, and the experiences of customers are determined to some extent by the employees. The interaction process can improve customer–employee fit, which then increases the efficiency and effectiveness of the service according to social dynamics theory (Hong et al., 2020). Customers become more knowledgeable about EVs as the interaction proceeds, which makes it easier for them to reach the cognitive state of being convinced and prepared to purchase. They also perceive the salespeople’s enthusiasm and expertise as they converse with them about EVs (Marinova et al., 2017). Positive interactions help customers to develop a closer bond of intimacy and trust with salespeople, which increases EEV. In order to deliver a service that is more specifically tailored to the customer’s demands, salespeople promptly solicit feedback from the customer (Su et al., 2007). Customers will also strengthen their SEV because their psychological needs are satisfied, and their feedback is adopted. Thus, the following hypotheses were addressed in this study.

*H2a*: SCI has a positive effect on FEV.

*H2b*: SCI has a positive effect on EEV.

*H2c*: SCI has a positive effect on SEV.

#### 2.3.2. Impact of EPV on the intention to purchase EVs

Along with the consumption upgrading and the product quality demand, the EPV is given a lot of attention. Many studies have provided empirical evidence to support the view that EPV significantly affects consumer purchasing intention and behavior. The value of shopping is provided by the complete shopping experience rather than simply purchasing the product (Pantano et al., 2021). Studies have demonstrated that purchasing behaviors do not always depend on economic rationality; instead, they are primarily motivated by emotions (Groth, 2016). Sensorial and emotional experience increases the value of the use of a product and influences customer satisfaction and the intention in shopping behavior (Japutra et al., 2021; Gulzari et al., 2022). Customers’ purchasing intentions (PIs) will be somewhat influenced by their sense of self-worth, social identity, pleasure, and comfort. Thus, this study assumes that the FEV, EEV, and SEV will encourage customers to purchase EVs.

*H3a*: FEV has a positive effect on the intention to purchase EVs.

*H3b*: EEV has a positive effect on the intention to purchase EVs.

*H3c*: SEV has a positive effect on the intention to purchase EVs.

#### 2.3.3. The mediating role of EPV

EPV is created during an interaction and then influences customers’ choices. Kim and Choi (2013) found that the service outcome and interaction quality significantly affect customers’ experience. In addition, when customers acquire more knowledge and skills about EVs *via* interactions, their functional experience is more valuable, and they are more likely to purchase EVs (Wang et al., 2018). The customer’s EEV in the EV purchasing process is the satisfaction of needs related to identity, belonging, and connection (Shen et al., 2016). Combined with attracting customers, interaction in selling EVs can gradually create emotional resonance. This process facilitates the acceptance of the conveyed messages, including the car company’s spiritual concept, leading to EV purchases. Thus, the following hypotheses were proposed.

*H4a*: EPV plays a mediating role in the effect of ECI on the intention to purchase EVs.

*H4b*: EPV plays a mediating role in the effect of SCI on the intention to purchase EVs.

#### 2.3.4. Different business models

Car companies with diverse business models have different interactions with their customers. Most traditional car companies with multi-business models have relatively remote dealerships in suburban areas (Cenglin, 2012). They generally lack to consider how to improve the arrangement, lighting, fragrance, interior displays, and other aspects of the selling environment. According to previous studies, the poor profit margin of EVs, the unavailability of EV models in sales outlets, and the high time cost of marketing EVs make traditional car companies frequently lack the initiative to offer EVs to customers (Matthews et al., 2017). In addition, the apps provided by numerous multi-business automobile companies give a terrible user experience with little or no customer usage, and they do not engage with customers anywhere and anytime.

By contrast, customers of car companies with a single-business model get experiences from four aspects: products, services, digital contacts, and lifestyle (Yang et al., 2021). These experiences help customers develop a deep brand relationship with car companies. The most typical example is Tesla, which chose a direct-sales model in which its vehicles are sold at fixed prices online or through factory-owned stores and service centers (Cahill et al., 2014). In addition, this car company leads customers to aspire to intelligent driving technologies, which provide customers with a better experience. Thus, the following hypothesis was addressed in this study.

*H5a*: There are differences in the impacts of interactions on the EPV and PI for car companies with different business models.

## 3. Methods and materials

### 3.1. Methods

Partial least squares structural equation modeling (PLS-SEM) combines principal component analysis with multiple regression analysis, and it has advantages when dealing with complex causal relationships. In this study, we adopted PLS-SEM because of the following reasons. First, the PLS-SEM model can handle the problem of non-normally distributed data. In contrast, the classic covariance-based structural equation modeling (CB-SEM) assumes that all observations follow a multivariate normal distribution. The sales contract, experience value, and purchasing intention observations used in this study did not follow a normal distribution. Thus, the CB-SEM cannot be used to analyze the variables. Second, ECI and SCI are second-order formative variables, and the CB-SEM cannot handle formative indications. Third, we aimed to integrate hypothesis testing to determine how interactions affected the intention to purchase EVs through experience value according to the SOR model. Therefore, the PLS-SEM approach was a suitable choice for evaluating the factors that might influence the intention of customers to purchase EVs in this study.

### 3.2. Materials

A questionnaire was used as a measurement tool in this study, and the design of it came from questionnaires successfully used in previous studies (see Appendix Table A.1). Before the formal distribution of the questionnaire, the authors selected 20 colleagues or friends who were about to purchase an EV for a pre-survey. Some minor linguistic adjustments and modifications were made to ensure that the participants could comprehend the meaning of each question. We selected six cities (Beijing, Shanghai, Tianjin, Chongqing, Shenzhen, and Hangzhou) to distribute the questionnaire, and they belong to the national EVs promotion demonstration cities. These cities have the following advantages compared to other cities in China: First, there are more EV sales stores and complete supporting facilities. Second, EVs have more online promotion and diversified sales methods in these cities. Third, more potential customers in these cities have experienced EVs online and offline. Questionnaire Star is a professional questionnaire distribution company that we commissioned to distribute online questionnaires in these six cities. At the same time, we went to sales stores in these cities and invited customers to scan the QR (Quick Response) code and fill in the questionnaire.

The target population is those who have had a complete online and offline car viewing or purchasing experience with a car company within 6 months. The complete online and offline experiences include browsing information about EVs on social media and car company apps, seeing EVs in a sales store, and having a test drive experience. Therefore, we set questions such as “Have you seen electric vehicles in an offline sales store within six months?” to find the target population. In the end, we collected 4,485 questionnaires (including 2,871 online and 1,614 offline questionnaires), leaving 1,014 valid questionnaires after eliminating invalid ones. According to Hair et al. (2011), minimum sample size should be equal to the larger of the following: (1) ten times the largest number of formative indicators used to measure one construct or (2) ten times the largest number of structural paths directed at a particular latent construct in the structural model. Therefore, the sample size of this study needs to be larger than 60 and 1,014 meets the requirements. The socio-demographic characteristics of participants are reported in Table 1. In order to know what kind of business model the car company is, we provided a selection of the 16 top-selling electric car companies in China for participants and a fill-in-the-blank item. If the 16 options did not contain the car company the participant expected, participants could fill in the blank item with the company they expected. After that, we judged which business model one car viewing experience belongs to according to the car company.

**Table 1:** Sample information (*N* = 1,014).

Demographics		Frequency	Percentage
Gender	Male	611	60.3
	Female	403	39.7
Age	18–25	159	15.7
	26–30	366	36.1
	31–35	281	27.7
	36–45	142	14
	46–55	57	5.6
	55 and over	9	0.9
Education	Middle School and below	21	2.1
	High School	121	11.9
	Junior college	248	24.5
	Undergraduate	561	55.3
	Postgraduate and above	63	6.2
Family annual income	Less than ¥100,000	122	12
	¥100,000–¥200,000	398	39.3
	¥200,000–¥300,000	314	31
	¥300,000–¥400,000	107	10.6
	¥400,000–¥500,000	30	3
	More than ¥500,000	43	4.2

Furthermore, the participants rated their level of agreement for several questions on a scale from 1 (strongly disagree) to 7 (strongly agree). Regarding the survey architecture development, the questions were randomized for each participant to reduce question order bias. In contrast, some questions with a defined response were inserted to avoid attention issues during the investigation.

## 4. Results

Statistical analyses of the data collected in this study were conducted using SPSS 26 and PLS-SEM 3. The data were examined to assess the sampling adequacy (KMO test) and data normality (Bartlett’s test of sphericity). The KMO score was 0.927, which exceeded the threshold of 0.70. Bartlett’s measure was also highly significant (*p* < 0.001). Thus, the data did not conform to the assumption of the independence of variables. The data collected using the questionnaire were well-structured and appropriate for factor analysis. The PLS-SEM model evaluation process had two stages. The outer model was first examined and assessed by its type. In the case of reflective measurement models, it is necessary to assess the reliability, convergent validity, and discriminant validity of indicators and constructs. For formative measurement models, it is necessary to assess the significance and relevance of the external weights of the indicators and multicollinearity. Thus, the capacity of each measurement indicator to explain the variables was determined. In the second stage, the inner model was examined by focusing on the path coefficients, the explanatory ($R^{2}$), and the predictive ($f^{2}$) capabilities. The effects of the interactions between car companies and customers on the intention to purchase EVs under different business models were then examined using multiple group analysis (MGA-PLS).

### 4.1. Outer model analysis

Some mature scales were contextualized in constructing the outer model, and certain variables were not considered in previous research. For this reason, it was essential to verify the questionnaire’s validity before assessing the outer model’s reliability. The consistency of the test items inside the variables was assessed based on the composite reliability (CR) and Cronbach’s alpha (Cronbach’s α). It is generally accepted that CR > 0.7 and Cronbach’s α > 0.7 indicate that the observed items are strongly linked with the variables.

In the outer model, measures used to assess the same latent variable are located at the same latent factor level, known as the outer model’s convergent validity. Standardized factor loadings for the observed indicators >0.5, average variance extracted (AVE) > 0.5, and CR > 0.7 are typically used to measure the convergent validity (Fornell and Larcker, 1981). As shown in Table 2, the AVE values ranged from 0.604 to 0.877, and the standardized factor loadings for all 28 assessed questionnaire items were 0.757, demonstrating the high convergent validity of the variables.

**Table 2:** Model results of SFL, CA, CR, and AVE.

Variable	Item	Standardized factor loading (SFL)	Cronbach’s Alpha (CA)	Composite reliability (CR)	Average variance extracted (AVE)
ECI_ON	ECI_ON1	0.784	0.796	0.867	0.620
	ECI_ON2	0.776			
	ECI_ON3	0.795			
	ECI_ON4	0.795			
ECI_OF	ECI_OF1	0.814	0.697	0.832	0.623
	ECI_OF2	0.786			
	ECI_OF3	0.766			
SCI_PD	SCI_PD1	0.838	0.760	0.862	0.676
	SCI_PD2	0.816			
	SCI_PD3	0.811			
SCI_VB	SCI_VB1	0.780	0.796	0.867	0.620
	SCI_VB2	0.781			
	SCI_VB3	0.799			
	SCI_VB4	0.789			
FEV	FEV1	0.763	0.781	0.859	0.604
	FEV2	0.801			
	FEV3	0.757			
	FEV4	0.787			
EEV	EEV1	0.946	0.954	0.966	0.877
	EEV2	0.931			
	EEV3	0.940			
	EEV4	0.929			
SEV	SEV1	0.933	0.921	0.954	0.875
	SEV2	0.927			
	SEV3	0.945			
PI	PI1	0.901	0.884	0.928	0.812
	PI2	0.899			
	PI3	0.903			

ECI_ON, online environment-customer interaction; ECI_ON1, the first item in the online environment-customer interaction; ECI_OF, offline environment-customer interaction; SCI_PD, product interaction in salesman-customer interaction; SCI_VB, verbal interaction in salesman-customer interaction; FEV, functional experience value; EEV, emotional experience value; SEV, social experience value; PI, purchasing intention.

The cross-loading matrix (cross-loading) and the Fornell–Larcker criterion demonstrated the discriminate validity of the SEM. Appendix Table B.1 shows that the variable’s factor loading for each questionnaire item was higher than its factor loading for the other variables (factor loading > cross-loading). In addition, the results of Table 3 indicated that the AVE value of each variable exceeded the square root of the correlation between the variable and the other variables, following the Fornell–Larcker criterion (Fornell and Larcker, 1981).

**Table 3:** Discriminant validity and the correlations.

	ECI_ON	ECI_OF	SCI_PD	SCI_VB	FEV	EEV	SEV	PI
ECI_ON	0.787							
ECI_OF	0.604	0.789						
SCI_PD	0.566	0.578	0.822					
SCI_VB	0.560	0.637	0.665	0.787				
FEV	0.551	0.531	0.600	0.614	0.777			
EEV	0.129	0.138	0.147	0.256	0.113	0.936		
SEV	0.110	0.128	0.129	0.194	0.106	0.864	0.935	
PI	0.294	0.300	0.367	0.385	0.411	0.067	−0.015	0.901

The diagonal is the root value of AVE, and the lower triangle is the Pearson correlation.

ECI_ON, online environment-customer interaction; ECI_ON1, the first item in the online environment-customer interaction; ECI_OF, offline environment-customer interaction; SCI_PD, product interaction in salesman-customer interaction; SCI_VB, verbal interaction in salesman-customer interaction; FEV, functional experience value; EEV, emotional experience value; SEV, social experience value; PI, purchasing intention.

Formative variables were assessed to determine the validity of the indicators with outer weights >0.2. The significance was calculated for the coefficients of the indicators (t > 1.96 and *p* < 0.01) using the bootstrap resampling method by drawing 5,000 samples. The outer weights were more than 0.4 for each indicator, as shown in Table 4, and were statistically significant (*p* < 0.01). The issue of covariance among formative indicators was also considered, i.e., variance inflation factor (VIF) < 3.3 (Thongrattana, 2010). The two formative variables related to SCI and ECI had VIFs of 1.956 and 2.334, respectively, which were below the threshold of 3.3 and were not affected by the covariance issue.

**Table 4:** Outer weights of interaction construct and their significance.

	Outer weights	STDEV	|O/STDEV|	*p* value
ECI_ON - > ECI	0.589	0.072	8.152	0.000
ECI_OF - > ECI	0.529	0.075	7.025	0.000
SCI_PD - > SCI	0.414	0.069	5.956	0.000
SCI_VB - > SCI	0.676	0.068	9.947	0.000

ECI_ON, online environment-customer interaction; ECI_ON1, the first item in the online environment-customer interaction; ECI_OF, offline environment-customer interaction; SCI_PD, product interaction in salesman-customer interaction; SCI_VB, verbal interaction in salesman-customer interaction; STDEV: standard deviation.

### 4.2. Inner model analysis

The PLS algorithm approach was utilized to assess the fits of the explanatory factors to the predictions of the outcome variables. Bootstrap iterative sampling was used to draw 5,000 samples for computing and evaluating the parameters relevant to the model coefficients. The results indicated that FEV, EEV, and SEV had $R^{2}$ values of 0.48, 0.39, and 0.31, respectively. Thus, interactions predicted FEV more accurately than EEV and SEV values. The effect size ($\;f^{2}$) has a minimum cutoff value of 0.02, which was used to quantify the impact of eliminating a specific latent variable on the endogenous variable. According to Table 5, the indicator of the predictive effect of the explanatory variable ECI on the outcome variable FEV was 0.066, and thus, higher than the minimum threshold. The indications of the predictive effects of SCI on FEV, EEV, and SEV ranged from 0.035 to 0.210, and all were higher than the minimum threshold. The predictive effects of FEV, EEV, and SEV on purchase intention are in the range of 0.028 to 0.207, which is a good prediction effect. Cross-validation was conducted (Henseler, 2009) to further assess the stability and fitness of the model, and the results ranging from 0.045 to 0.816 demonstrated the model’s validity. Additionally, the overall model standardized root mean square residual (SRMR) of 0.059 satisfied the requirement of SRMR <0.08 (Henseler et al., 2016), demonstrating the model’s fitness.

**Table 5:** Structural model results and effects sizes ($f^{2}$).

Criterion variable	Predictor variable	H	Path coefficient	STDEV	T	*f* ^2^	Conclusion
FEV	ECI	H1a	0.265^***^	0.050	5.320	0.066	Support
	SCI	H2a	0.475^***^	0.053	8.881	0.210	Support
EEV	ECI	H1b	−0.038	0.044	0.860	0.001	Not Support
	SCI	H2b	0.261^**^	0.047	5.544	0.035	Support
SEV	ECI	H1c	−0.001	0.043	0.023	0.000	Not Support
	SCI	H2c	0.186^***^	0.046	4.061	0.018	Support
PI	FEV	H3a	0.411^***^	0.041	10.090	0.207	Support
	EEV	H3b	0.281^***^	0.079	3.534	0.025	Support
	SEV	H3c	0.301^***^	0.076	3.962	0.028	Support

*p < 0.10. **p < 0.05. ***p < 0.010. Pearson correlation, two-tailed.

ECI, environment-customer interaction; SCI, salesman-customer interaction; FEV, functional experience value; EEV, emotional experience value; SEV, social experience value; PI, purchase intention; STDEV, standard deviation.

The following results can be summarized based on Table 5. First, the path coefficients of 0.265 and 0.475, respectively, confirmed hypotheses H1a and H2a by showing that ECI and SCI had considerable positive effects on FEV. SCI was more effective than ECI regarding how much each variable influenced FEV. The effect of ECI on EEV and SEV failed to pass the significance test. By contrast, the beneficial impacts of SCI on EEV and SEV were more significant, with path coefficients of 0.261 and 0.186, respectively, thereby supporting hypotheses H2b and H2c. FEV, EEV, and SEV significantly affected the purchasing intention, with path coefficients of 0.411, 0.281, and 0.301, respectively, thereby supporting hypotheses H3a, H3b, and H3c.

### 4.3. Intermediary testing

The mediation effect test protocol was employed to evaluate the mediating effect of EPV (Zhao et al., 2010). In total, 5,000 samples were used, and the significance of the mediating impact was determined by assessing whether the 95% confidence interval of the indirect effect included 0. According to the results (see Table 6), the EPV was a significant mediating factor for the effect of ECI on PI. The mediating effects of FEV, EEV, and SEV were all significant, with indirect effects of 0.339, 0.067, and 0.069, respectively. The overall effect of EPV on mediating the effect of SCI on PI was also significant. All three EPV types had significant mediating effects. However, the mediating effect of FEV was smaller than before, with 0.277, whereas the effects of EEV and SEV were more significant than those under ECI, with 0.086 and 0.083, respectively. The amplitudes of the path impacts of the three mediating factors were then compared. Compared with the mediating effects of ECI and SCI on PI, the mediating effect of FEV was much more significant than those of EEV and SEV.

**Table 6:** Bootstrap analysis of the intermediate effects test.

	ECI → EV → PI			SCI → EV → PI		
Mediating effect	Effect	95% confidence intervals		Effect	95% confidence intervals	
		Lower	Upper		Lower	Upper
FEV	0.339	0.232	0.462	0.277	0.160	0.408
EEV	0.067	0.026	0.126	0.086	0.028	0.157
SEV	0.069	0.028	0.129	0.083	0.036	0.151
Total effect	0.475	0.286	0.717	0.446	0.224	0.716
Comparison of mediating effects						
FEV/EEV	0.272	0.141	0.399	0.191	0.042	0.338
FEV/SEV	0.270	0.293	0.489	0.194	0.056	0.347
EEV/SEV	−0.002	−0.023	0.110	0.003	−0.042	0.134

ECI, environment-customer interaction; SCI, salesman-customer interaction; FEV, functional experience value; EEV, emotional experience value; SEV, social experience value; EV, experience value; PI, purchasing intention.

### 4.4. Multi-group analysis

The Multi-group analysis is used to determine the presence and significance of differences by estimating the parameters (path coefficients) for predefined groups within a sample (Hair et al., 2017). If the value of p for the difference in the group path coefficients was less than 0.05 or more than 0.95, a parametric test and the MGA-PLS approach are needed to identify significant differences (Hair et al., 2014). We used this technique to investigate how different business models were affected by ECI and SCI.

According to the results obtained by multi-group analysis (Table 7), the effects of ECI and SCI on PI varied greatly under two business models. The effect of ECI on FEV was considerable under single and multiple business models, but the single-business model had a more substantial positive effect. Under both business models, the impacts of ECI on EEV and SEV were negative or insignificant. In addition, SCI significantly increased FEV, EEV, and SEV under the single-business model. By contrast, SCI only significantly increased FEV under the multi-business model. The effect of SCI on EEV varied greatly depending on the model. EEV and SEV only passed the test under the single-business model, and the difference between the effects of the two models was significant. The effect of FEV on the intention to purchase EVs was significant under both business models.

**Table 7:** Impact of environmental and interpersonal interactions on purchase intention of EVs under different business models.

	Path Coefficients (single)	STDEV (single)	t (single)	Path Coefficients (multiple)	STDEV (multiple)	t (multiple)
ECI - > FEV	0.375^***^	0.071	5.269	0.184^***^	0.056	3.281
ECI - > EEV	−0.251^***^	0.065	3.863	0.101	0.066	1.543
ECI - > SEV	−0.112	0.063	1.787	0.073	0.062	1.170
SCI - > FEV	0.395^***^	0.069	5.727	0.541^***^	0.063	8.622
SCI - > EEV	0.484^***^	0.068	7.145	0.124	0.067	1.857
SCI - > SEV	0.293^***^	0.066	4.456	0.114	0.065	1.760
FEV - > PI	0.427^***^	0.055	7.823	0.401^***^	0.055	7.322
EEV - > PI	0.493^***^	0.111	4.435	0.154	0.105	1.464
SEV - > PI	0.514^***^	0.104	4.958	0.182	0.103	1.765

ECI, environment-customer interaction; SCI, salesman-customer interaction; FEV, functional experience value; EEV, emotional experience value; SEV, social experience value; PI, purchasing intention.

## 5. Discussion

The intention to purchase EVs is crucial for anticipating the market size and consumption patterns. This study obtained sufficient theoretical and empirical support for the classification of interactions based on two dimensions: ECI and SCI. These two dimensions are based on the connotations and dimensions of interactions between car companies and customers in two business models. In addition, our findings provide new insights into how salespeople traits and online market circumstances affect the final effect of interactions on the purchase of EVs.

### 5.1. General discussion

In Section 4, we showed that there is an impact of ECI and SCI on EPV, so we further discussed the impact of each of the two dimensions of ECI (online ECI and offline ECI) and SCI (PD and VB) on EPV. The results obtained in this study demonstrated (see Appendix Table C.1) that online ECI had a significant impact on FEV (β=0.156,ρ<0.01). This finding indicates that customers’ functional needs were satisfied, and they could access detailed information (such as usage instructions) through the official website and the car company’s app. Nöjd et al. (2020) also found that digital technology can support customers’ desires and enhance their experience. However, online and offline ECI had little effect on EEV and SEV, possibly because it takes longer to nurture and instill emotional and social values. In addition, ECI is less adaptable than SCI at detecting customers’ emotional states, preventing customer unhappiness, and boosting customer contentment. SCI had beneficial effects on FEV (β=0.197,ρ<0.01), EEV (β=0.108,ρ<0.01), and SEV (β=0.077,ρ<0.01), which suggested that experiencing EVs could effectively help them to understand the benefits of EVs. This process reduced their psychological anxieties, increased their enjoyment, and raised their environmental consciousness about purchasing an EV. Similarly, Shen et al. (2016) demonstrated the importance of co-creating PD with customers. VB had the best effect on FEV (β=0.321,ρ<0.01), EEV (β=0.176,ρ<0.01), and SEV (β=0.126,ρ<0.01). Thus, developing online and mobile platforms can significantly improve customers’ understanding of EVs, but salespeople still play a crucial role in the interaction process. The salespeople’s technical expertise and service attitude can significantly influence the customer’s perception and PI. Higueras-Castillo et al. (2019) believed that the sale force should provide more information explaining the product performance. Gerardo et al. (2018) also found that the most significant factors that influenced the purchasing intentions of prospective purchasers were the salespeople’s enthusiasm and their knowledge of EVs.

In addition, SEV, EEV, and FEV significantly impacted the intention to purchase EVs. It indicates that recognition of the functional value of EVs, a positive emotional experience of viewing a car, and social recognition can increase the willingness to purchase an EV. A good functional experience enhances the practical and functional value of users, thus increasing their purchasing intention; while a good emotional experience enhances the enjoyment and emotional value of users, thus also increasing their purchasing intention (Yang et al., 2021). Furthermore, SEV, EEV, and FEV had mediating roles in the impacts of interactions on the intention to purchase an EV. The mediating effect of FEV was much more significant than those of EEV and SEV. Therefore, the more FEV customers generate during interactions, the more likely they are to purchase an EV.

When considering the business models, the results are further discussed. The positive effect of ECI on FEV was more significant for single-business model car companies. This result supports the phenomenon that not all car companies can successfully promote their products through live events or other online activities (Habich-Sobiegalla et al., 2019). Many car companies with multi-models face problems such as underdeveloped mobile platforms and amateurish Internet sales platforms. The impact of ECI on EEV for single-business model companies is negative. These companies, such as BYD, Tesla, and Xiaopeng, account for most EV sales in the Chinese market. However, they generally have problems such as long pickup cycles and uncertain waiting times. These single-business model car companies often offer a complete online car purchase service that displays an estimated delivery date after the customer has selected their desired car. Some have delivery dates as high as 3 to 5 months, which inevitably leads to a bad experience. In our sample, BYD, Tesla, and Xiaopeng accounted for 34.5, 16, and 8.9% of the sample. Therefore, people in the sample who had experienced or purchased EVs from these car companies should be aware of this pickup cycle problem. The salespeople often deliberately avoid informing customers about the long vehicle delivery lead time, so this factor played a minor role in the impact of SCI on EEV. At the same time, most of the apps of single-business model car companies integrate a variety of segments such as car purchase, car use, car maintenance, social, and e-commerce. Most customers only use a few segments, and too many segments can affect customers’ browsing experience. In addition, some car companies deliberately filter out negative comments in the community section of the app. Too many positive or praising comments could make customers question the platform’s authenticity and create resistance. All of these are reasons why ECI has a negative impact on consumers’ EEV. The effects of SCI on EEV and SEV failed the test for the multi-business model car companies, indicating that the salespeople in these companies did not aggressively guide customers to concern EVs. They also did not fully promote the social symbol of driving EVs, such as pro-environment identification. These barriers made it challenging for customers to acquire knowledge of EVs following interactions. Thus, they did not feel satisfied and happy since the product would meet their expectations. This explains why EEV and SEV did not significantly affect PI in the multi-business model company.

### 5.2. Practical implications

Given the different effects of sales interactions for car companies with two business models, some suggestions are provided. First, to maximize the experience value, car companies could continuously update and develop the functions of the online mobile platforms and strive to provide timely and convenient interactions to customers. Before purchasing an EV, customers can require comprehensive and adequate information (such as high-tech features) from the automobile industry. Second, it is necessary to increase the professionalism of salespeople by enhancing their knowledge and expertise in marketing EVs. Salespeople should be able to respond to customers’ questions about EVs. In addition, they should present a positive outlook on purchasing an EV by emphasizing the prosocial qualities and environmental benefits. This can enhance the customers’ recognition and correct their ingrained perceptions of EVs. Last, the online ECI of single-business car companies has not been able to meet customers’ emotional and social needs; so, some online services should be improved. Car companies could analyze the different categories of customers and tailor the style and contents of their interactions to meet their specific needs. For example, push customized content for different groups on the app.

The results in this study can provide a basis and reference for policymakers to develop EV-promoting strategies. First, given that some customers lack sufficient knowledge about EVs, the government could actively promote and popularize EV-related information (such as vehicle performance, environmental benefits, and high-level intelligence) on digital platforms. This measure could encourage society to purchase EVs and foster green travel concepts. Second, to ensure customers have a thorough and understandable grasp of EV incentive policies, the government could present EV-related policies in a form that customers can comprehend on the leading mobile platforms. Third, more chances can be provided for customers to get in touch with EVs. For example, the government could encourage car companies to organize face-to-face activities of EVs in the experience center. This can make potential customers gain a deeper understanding and experience of EVs through interactions (such as explanations by salespeople and test drives). Finally, the government could guide the training of specific EV-related employees and accelerate the transformation of car salespeople. They should be able to comprehend the benefits and traits of EVs and encourage customers to have faith in EVs.

## 6. Conclusion and future research

The interaction between sellers and customers is significant for promoting EVs but generally attaches little attention in previous studies. In this study, we explored how interactions influence the intention of customers to purchase EVs. We also identified the differences in the interactions among car companies with two business models. The following results were obtained by modeling the data collected from a large-scale survey in China. First, ECI had a positive effect on FEV but no significant effects on EEV and SEV. SCI had a positive effect on three dimensions of EPV. Second, FEV, EEV, and SEV all positively affected PI. Finally, we looked at interactions of two business model car companies. ECI of single-business model car companies had a significant positive effect on FEV, but there was a significant negative effect on EEV and SEV. However, the impact of SCI on FEV, EEV, and SEV for single-business model car companies is positive. Furthermore, the ECI and SCI of multi-business model car companies positively affected FEV, but the effects on EEV and SEV were insignificant. The study revealed the effect of interactions by car companies with different business models and discussed the challenges, opportunities, and emerging trends in China’s EV market. It provides a new perspective for EV car companies and the government to promote EVs after the subsidy policy has been withdrawn.

We obtained some insightful results in this study, but further studies are still needed. We only used data from China, which could limit the broader application of our results. Future studies could use data from more countries to confirm the reliability of our results. In addition, the sample data used for the analysis in this study were cross-sectional or static data from a specific period. Thus, it was impossible to detect changes in customers’ intentions to purchase EVs over time. In conclusion, a follow-up study could use a longitudinal design method with regular monitoring and validation to produce more accurate and scientific results.

## Data availability statement

The original contributions presented in the study are included in the article/Supplementary material, further inquiries can be directed to the corresponding author.

## Author contributions

WL and RL developed the original idea for this article. XC designed the experiment. WL and MW analyzed the results and wrote the paper. All authors have read and agreed to the published version of the manuscript.

## Funding

This study was supported by grants from the National Natural Science Foundation of China (Nos. 71904067, 72274083, and 72104108).

## Conflict of interest

The authors declare that the research was conducted in the absence of any commercial or financial relationships that could be construed as a potential conflict of interest.

## Publisher’s note

All claims expressed in this article are solely those of the authors and do not necessarily represent those of their affiliated organizations, or those of the publisher, the editors and the reviewers. Any product that may be evaluated in this article, or claim that may be made by its manufacturer, is not guaranteed or endorsed by the publisher.

## Supplementary material

The Supplementary material for this article can be found online at: https://www.frontiersin.org/articles/10.3389/fpsyg.2023.1129752/full#supplementary-material
