# Supplementary material for: The impact of interaction on the adoption of electric vehicles: Mediating role of experience value
Source: Front Psychol. 2023 Feb 28;14:1129752. doi: 10.3389/fpsyg.2023.1129752 (PMC10011173; doi:10.3389/fpsyg.2023.1129752)
Supplement: Supplementary file 1 [file Data_Sheet_1.pdf]

Appendix Table A.1 Measurement question items

		Measurement items		References
Environment-customer interaction	ECI	Online ECI (ECI_ON)	1. This car company can attract my attention to EVs through social media such as Weibo and video websites.（ECI_ON1）	Chen（2022）<br>Harvinder Singh（2014）<br>Bitner（1992）
			2. The APP or official website of this car company covers comprehensive services, which can meet my basic needs to understand or buy EVs.（ECI_ON2）	
			3. The APP or official website launched by the car company made me feel relaxed and happy with its unique page layout, background music and novel short films.（ECI_ON3）	
			4. The APP module launched by the car company is reasonably designed and convenient to use.（ECI_ON4）	
		Offline ECI (ECI_OF)	1. Sales stores are created through layout and details, such as lighting and music to create a comfortable shopping atmosphere.（ECI_OF1）	
			2. The sales store is clean and tidy.（ECI_OF2）	
			3. The sales store is in a convenient transportation.（ECI_OF3）	
Salesman-customer interaction	SCI	Product interaction (SCI_PD)	1. In the process of test drive, you can feel the intellectualization and technology of EVs. (SCI_PD1)	Parasuraman（1988）<br>Chen（2022）<br>Hooper（2013）
			2. During the test drive, you can feel the improvement of the driving experience of EVs, such as driving tranquility and acceleration performance. (SCI_PD2)	
			3. The test drive process changed my overall understanding of EVs. (SCI_PD3)	
		Verbal interaction (SCI_VB)	1. The salespeople were warm and friendly and offered to sell me EVs.	

		(SCI_VB1)<br>2. The salespeople have a positive attitude towards the use and development prospects of EVs. (SCI_VB2)<br>3. The salespeople can give a comprehensive and professional explanation to my questions about EVs. (SCI_VB3)<br>4. The salespeople listen to my needs when providing services and adjust accordingly. (SCI_VB4)	
Functional experience value	FEV	1. Through the interaction, I learned knowledge and skills related to EVs. (FEV1)<br>2. Through the interaction, I think EVs are more practical. (FEV2)<br>3. Through service interaction, I think I can more easily learn how to use electric cars. (FEV3)<br>4. Through the interaction, I think the company's EVs are better than similar EVs. (FEV4)	Shen（2016）<br>Sweeney（2001）
Emotional experience value	EEV	1. The interaction process is full of fun. (EEV1)<br>2. The process of interaction made me feel relaxed and comfortable. (EEV2)<br>3. The process of interaction attracts my attention to the EVs. (EEV3)<br>4. I was satisfied with the overall process of interaction. (EEV4)	Babin（1994）<br>Holbrook（2006）<br>Rintama¨ki（2006）
Social experience value	SEV	1. During the interaction, I felt that I was being respected. (SEV1)<br>2. The interaction made me realize that using EVs can gain more social recognition. (SEV2)<br>3. I would like to share my experience of interaction with my friends/acquaintances about EVs. (SEV3)	Shen（2016）<br>Sweeney（2001）<br>Rintama¨ki（2006）
Purchasing intention	PI	1. I would prefer to purchase an EV over a conventional fuel car. (PI1)<br>2. I would like to purchase an EV soon. (PI2)<br>3. I would recommend electric cars to my friends and family. (PI3)	Petrick& Dubinsky(2003)<br>Cronin（2000）

Appendix Table B.1 Cross loadings

	ECI_ON	ECI_OF	SCI_PD	SCI_VB	FEV	EEV	SEV	PI
ECI_ON1	**0.784**	0.440	0.433	0.390	0.407	0.053	0.070	0.181

ECI_ON2	**0.776**	0.503	0.433	0.464	0.439	0.183	0.127	0.240
ECI_ON3	**0.795**	0.510	0.467	0.456	0.456	0.081	0.067	0.254
ECI_ON4	**0.795**	0.447	0.449	0.452	0.433	0.087	0.081	0.249
ECI_OF1	0.498	**0.814**	0.465	0.503	0.436	0.100	0.087	0.207
ECI_OF2	0.501	**0.786**	0.484	0.546	0.427	0.154	0.140	0.280
ECI_OF3	0.428	**0.766**	0.418	0.459	0.392	0.072	0.074	0.224
SCI_PD1	0.475	0.488	**0.838**	0.566	0.479	0.077	0.068	0.319
SCI_PD2	0.450	0.474	**0.816**	0.576	0.497	0.196	0.149	0.324
SCI_PD3	0.472	0.463	**0.811**	0.494	0.504	0.087	0.101	0.258
SCI_VB1	0.447	0.523	0.537	**0.780**	0.502	0.195	0.156	0.301
SCI_VB2	0.443	0.529	0.505	**0.781**	0.485	0.240	0.186	0.293
SCI_VB3	0.473	0.496	0.559	**0.799**	0.506	0.182	0.125	0.340
SCI_VB4	0.399	0.460	0.492	**0.789**	0.441	0.190	0.146	0.277
FEV1	0.434	0.426	0.486	0.469	**0.763**	0.025	0.012	0.270
FEV2	0.412	0.390	0.482	0.485	**0.801**	0.167	0.152	0.409
FEV3	0.426	0.411	0.453	0.480	**0.757**	0.076	0.108	0.269
FEV4	0.443	0.425	0.443	0.477	**0.787**	0.074	0.053	0.319
EEV1	0.147	0.154	0.161	0.278	0.123	**0.946**	0.807	0.079
EEV2	0.101	0.112	0.115	0.203	0.065	**0.931**	0.827	0.043
EEV3	0.131	0.136	0.139	0.248	0.142	**0.940**	0.799	0.086
EEV4	0.091	0.107	0.126	0.213	0.078	**0.929**	0.811	0.033
SEV1	0.099	0.111	0.097	0.169	0.062	0.817	**0.933**	-0.058
SEV2	0.095	0.105	0.112	0.179	0.120	0.823	**0.927**	0.014
SEV3	0.112	0.139	0.148	0.194	0.114	0.789	**0.945**	-0.002
PI1	0.262	0.266	0.323	0.309	0.372	0.017	-0.058	**0.901**
PI2	0.265	0.271	0.331	0.352	0.367	0.077	0.017	**0.899**
PI3	0.267	0.275	0.337	0.380	0.371	0.088	0.003	**0.903**

Abbreviations: ECI_ON: online environment to customer interaction; ECI_ON1: the first item of online environment to customer interaction; ECI_OF: offline environment to customer interaction; SCI_PD: product interaction in salesman to customer interaction; SCI_VB: verbal interaction in salesman to customer interaction; FEV: functional experience value; EEV: emotional experience value; SEV: social experience value; PI: purchasing intention.

Appendix Table C.1 Results of Bootstrap iterative sampling metho

	Path coefficient	STDEV	T (|O/STDEV|)
ECI_ON -> FEV	0.156^{***}	0.036	4.341
ECI_ON -> EEV	-0.022	0.026	0.859
ECI_ON -> SEV	-0.001	0.025	0.023
ECI_OF -> FEV	0.14^{***}	0.032	4.393
ECI_OF -> EEV	-0.02	0.024	0.828
ECI_OF -> SEV	-0.001	0.023	0.023

SCI_PD -> FEV	0.197***	0.045	4.342
SCI_PD -> EEV	0.108***	0.023	4.727
SCI_PD -> SEV	0.077***	0.021	3.739
SCI_VB -> FEV	0.321***	0.039	8.128
SCI_VB -> EEV	0.176***	0.041	4.273
SCI_VB -> SEV	0.126***	0.036	3.49

Note: *p < 0.10. **p < 0.05. ***p < 0.010. Pearson correlation, two-tailed.

Abbreviations: ECI_ON: online environment to customer interaction; ECI_OF: offline environment to customer interaction; SCI_PD: product interaction in salesman to customer interaction; SCI_VB: verbal interaction in salesman to customer interaction; FEV: functional experience value; EEV: emotional experience value; SEV: social experience value; PI: purchasing intention.
